# Cognitive neurodynamics of affective disorders

**DOI:** 10.1007/s11571-026-10507-2

**Published:** 2026-07-23

**Authors:** Luigi Manfredi, Xinpeng Liu, J. Douglas Steele

**Affiliations:** 1 Division of Respiratory Medicine and Gastroenterology, School of Medicine, Ninewells Medical School, University of Dundee, Level 7, Corridor L, Mailbox 1, Dundee, DD1 9SY UK; 2 Division of Neuroscience, School of Medicine, University of Dundee, Dundee, DD1 9SY UK

**Keywords:** Motivational dynamics, Opponent-process theory, Control systems, Psychiatric illnesses, Homeostasis, Affective disorders

## Abstract

Severe and enduring psychiatric illnesses, including melancholia and bipolar illness, show prolonged deviations in motivation and mood yet lack a unifying account of their long-term dynamics. Solomon’s opponent-process theory provides a qualitative framework for short-timescale affective responses, composed of a fast stimulus-locked a-process opposed by a slower adaptive b-process. Here we evaluated whether these dynamics can be implemented as a computational homeostatic controller, and whether distinct clinical trajectories emerge as canonical failure modes of the same system. We formulated a tractable control-systems model with feedforward a- and b-processes, and examined its behaviour across minute-scale and month-scale regimes. Under “healthy” parameters, the model reproduced classical opponent-process responses. Altering only opponent-process gain and decay generated a prolonged downward drift, matching the clinical timescale and asymmetry of melancholia. Reducing damping within the same controller produced an endogenous underdamped oscillation with long period and phase asymmetry characteristic of bipolar illness. Together, these proof-of-principle simulations suggest that severe severe affective illnesses may be expressed as distinct dynamical regimes of a single motivational homeostat. This framework generates testable predictions and may facilitate experimental quantification of opponent-process recovery, damping, and gain as mechanistic markers of severe affective illnesses.

**Supplementary Information:**

The online version contains supplementary material available at 10.1007/s11571-026-10507-2.

## Introduction

Severe and enduring psychiatric illness (SEPI) relevant to the present study are unipolar depression (Parker 1996, Taylor and Fink 2006), and bipolar illness (Goodwin 2007) which are associated with a significant reduction in average life expectancy (Steele et al. 2025). Unipolar depression (with melancholia; ICD-11 6A71.3/4; 6A80.3) is dominated by persistent negative affect and psychomotor and motivational reduction, whereas bipolar illness (ICD-11 6A60.0/1; 6A60.6/7) is characterised by recurrent alternation between elevated and depressed states. Addiction is clinically distinct but mechanistically relevant because it provides a well-studied example of opponent adaptation and allostatic drift within affective-motivational systems (Koob 2013). The physiological mechanisms that drive long-timescale illnesses remain poorly understood limiting progress toward more effective interventions.

Current models of motivation are fragmented across disorders, timescales and levels of explanation. Bipolar disorder has been modelled with nonlinear oscillators and related dynamical-systems approaches (Nunes et al. 2022), melancholia with circadian and HPA-axis bistability or toggle-switch models (Mizrachi et al. 2023), normal affect with opponent-process theory (Solomon 1980), addiction with allostatic theory (Koob 2013), and short-timescale learning with temporal-difference reinforcement learning (Suveges et al. 2025). These accounts are informative, but they usually emphasize different abnormalities of a partly shared positive and negative valence system architecture rather than a common homeostatic controller (Cuthbert and Insel 2013).

At a physiological level, convergent evidence implicates overlapping cortico-striatal-limbic circuitry involved in reward processing, aversive learning, emotion regulation, and stress adaptation, together with slower endocrine and circadian modulators (Goodwin 2007, Mizrachi et al. 2023, Koob 2013, Cuthbert and Insel 2013, Phillips and Swartz 2014, Grande et al. 2016). The Research Domain Criteria (RDoC) framework likewise emphasizes dimensional dysfunction within shared neurobehavioral systems rather than wholly disorder-specific mechanisms (Cuthbert and Insel 2013, Cuthbert and Insel 2013). We therefore asked whether a parsimonious control system model could capture these common dynamical motifs while remaining agnostic about many cellular, developmental, and pharmacological sources of heterogeneity.

In general medicine, illnesses are understood as failures of homeostatic physiological control systems that normally maintain regulated variables within stable bounds (Hall and Hall 2021). The same principle is likely to apply to affective–motivational regulation, yet the field lacks a simple theory capable of generating the prolonged deviations characteristic of severe affective illnesses. Solomon’s opponent-process theory offers a natural foundation for effective motivational regulation because it formalizes behaviour at the interaction of a fast stimulus-locked a-process and a slower adaptive b-process (Solomon 1980).

The central aim of the present study was therefore not to propose a new controller class, but to test whether this established opponent architecture can be written as a quantitative control system that reproduces normal adaptation, allostatic drift, melancholic decline, and bipolar oscillation as distinct dynamical regimes. We tested two linked hypotheses: (i) Solomon’s opponent-process dynamics can be reproduced as an engineering control system, and (ii) melancholia and bipolar illness reflect distinct failure modes of a single motivational homeostatic controller.

## Materials and methods

Solomon and Corbit’s theory describes the affective response to a discrete cognitive–perceptual stimulus as the sum of a rapid *a*-process and a slower adaptive *b*-process. We translated this formulation into a computational control-system model using a standard delayed feedforward and low-pass element. The controller class itself is therefore not claimed as novel; the novelty lies in mapping opponent-process dynamics onto a minimal homeostatic architecture capable of generating healthy, addictive, melancholic, and bipolar trajectories within one framework.

The *a*-process was implemented as an immediate feed-forward response to the stimulus input. The *b*-process was generated by a delayed opponent stage with variable gain and slow decay, implemented using standard low-pass filtering components. The net motivational state was defined as the difference between these components, consistent with Solomon’s original formulation and with classical physiological controllers composed of stimulus-driven and adaptive elements.

### Model structure and physiological interpretation

Let $s\left(t\right)={A}_{0}\left[u\right(t-{t}_{0})$-$u(t-{t}_{0}-d)]$ denote a finite stimulus of amplitude *A₀*, onset ${t}_{0}$, and duration $d$. The primary process was defined as $a\left(t\right)=s\left(t\right)$, and the net affective state as $e\left(t\right)=a\left(t\right)-b\left(t\right)$. The opponent process $b\left(t\right)$ was generated as a delayed, scaled transformation of $a\left(t\right)$ passed through one or more low-pass stages, $b\left(t\right)=K\left[\right({h}_{1}*{h}_{2}*a\left)\right(t-\tau \left)\right]$, where *K* denotes opponent gain, $\tau$ the onset delay, and ${h}_{1}$ and ${h}_{2}$ adaptation kernels with short and long-time constants. In physiological terms, the *a*-process represents rapid stimulus-linked valuation or salience; *τ* represents recruitment delay of opponent mechanisms; *K* captures opponent strength; and the low-pass stages capture slower recovery, memory, and allostatic accumulation rather than any single anatomical loop (Solomon 1980, Koob 2013, Cuthbert and Insel 2013, Phillips and Swartz 2014).

For bipolar simulations, the same homeostatic-control framework was represented phenomenologically in second-order form, $\ddot{e}\left(t\right)+2\zeta \omega \dot{e}+{\omega }^{2} e\left(t\right)=0$, so that reduced damping $(\zeta<1)$ generated an underdamped oscillatory regime. This representation is a phenomenological approximation of slow episode-indexed adaptation dynamics, not a claim that mood episodes are governed by a single literal oscillator at the neuronal level (Nunes et al. 2022, Phillips and Swartz 2014).

Two timescales were modelled. For normal motivational dynamics, parameters replicated Solomon’s minute-scale responses observed experimentally in humans and animals. For illness simulations, the model was reparametrized using day-to-month timescales to reflect the clinical duration of melancholia and bipolar illness, in which deviations persist for months to years. A single architectural model was used across all simulations; differences between health and illness arose solely from changes in opponent-process gain or decay (unipolar) or reduced damping in the second-order system (bipolar). This mirrors physiological modelling, where parameter changes within one controller yield distinct failure modes, and physiological modelling, in which altered parameters within the same controller give rise to distinct forms of regulatory failure.

To aid interpretation, model amplitudes were scaled to conventional clinical ranges for affective illness. For melancholia trajectories, the magnitude of negative deviation was matched to the typical range of Hamilton Depression Rating Scale (HDRS) scores; for bipolar simulations, elevated states were scaled using approximately half the range of Young Mania Rating Scale (YMRS) values, reflecting the shorter duration and lower overall amplitude of mania relative to depression in naturalistic longitudinal studies. These scales informed qualitative matching only and were not used as empirical training targets.

Stimuli were implemented as finite-duration inputs of defined amplitude and onset. Simulations were performed using standard control-systems methods, with Simulink (MathWorks and Simulink 2025) used to generate the time-series outputs presented in the Results. The aim was to demonstrate the dynamical consequences of canonical parameter changes, rather than to optimise parameters or fit individual patient data. Accordingly, the present simulations are illustrative, and formal parameter identifiability will require future estimation against longitudinal clinical, behavioural, and neuroimaging data.

## Results

### Clinical benchmarks for model simulations

**Fig. 1:**
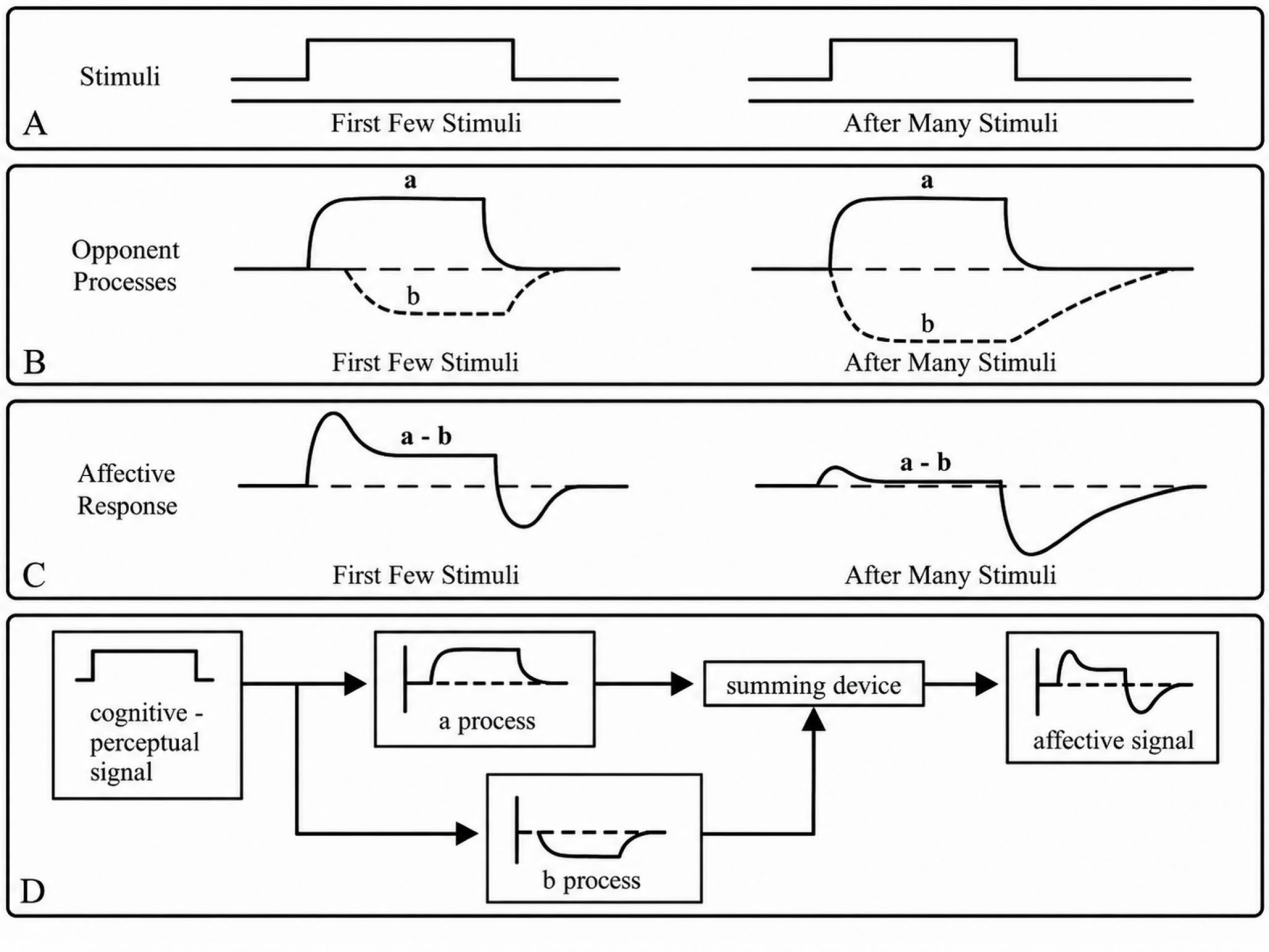
**A–C** Motivational dynamics in health described by Solomon & Corbit’s Opponent-Process Theory. This schematic illustrates the temporal evolution of motivational responses to a stimulus with repeated exposures. **A** The stimulus input is represented as a square pulse, identical in both early and repeated exposures. **B** The a-process (solid line) represents the immediate, stimulus-bound affective response and remains stable over time. The b-process (dashed line) emerges more slowly and increases in both amplitude and duration with repeated exposure. **C** The resulting net affective response (**a**, **b**) reflects the subjective emotional experience. With repeated stimulation, the opponent b-process becomes stronger and longer-lasting, leading to diminished pleasure during the stimulus and a more pronounced negative reaction once the stimulus ends. **D** Conceptual block diagram of the motivational system based on the Opponent-Process Theory. A cognitive-perceptual input signal generates parallel responses: a fast a-process and a delayed b-process. These are combined in a summing module to produce the final affective signal (**a** − **b**), which evolves over time in response to repeated stimuli and adaptation

**Fig. 2:**
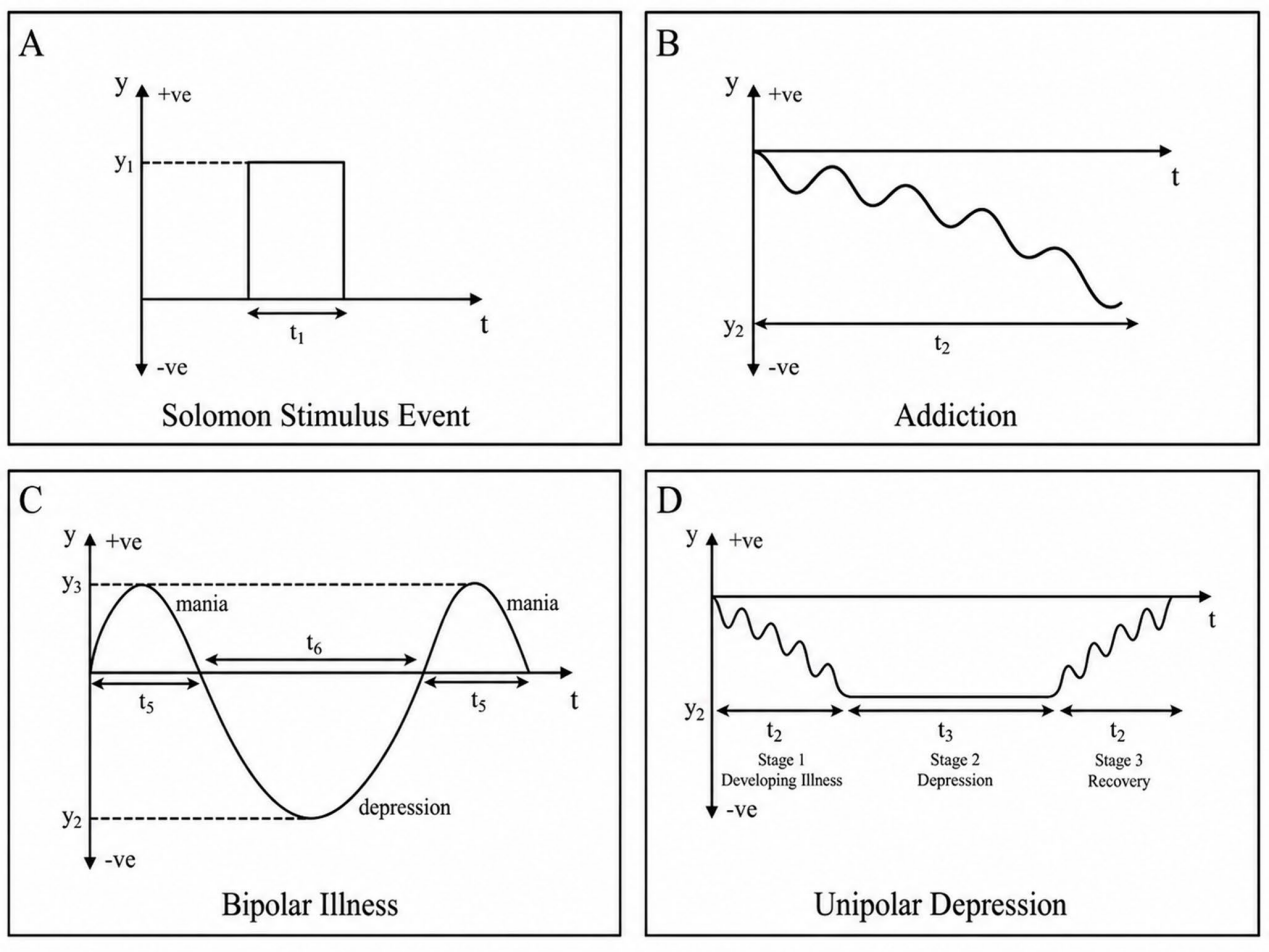
Definition of durations (t) and amplitudes (y) used in modelling. **A** cognitive event modelled as a stimulus of amplitude y_1_ and duration t_1_, representing a typical short-lived perceptual event. **B** Addiction: Repeated drug exposure before the increasing b-process has decayed to baseline, results in a cumulative downward drift, known as allostasis: total duration t_2_, negative baseline approaching y_2_. **C** Bipolar Illness: Oscillatory affective pattern between manic and depressive episodes. Manic phases peak at y_3_, depressive phases reach y_2_, with mania lasting t_5_ and depression lasting longer as t_6,_ with the oscillation repeating. **D** Unipolar Depression: Affective trajectory across three stages: illness development (t_2_), sustained depressive episode (t_3_), and recovery (t_2_). The lowest affective value reaches y_2_, with slow return to baseline. as recovery

The qualitative opponent-process framework and the clinical benchmark values used to calibrate the simulations are summarised in Figs. 1 and 2. Simulations were then calibrated to reproduce the characteristic durations and amplitudes of melancholia and bipolar illness (Fig. 2). In bipolar illness, the oscillatory trajectory alternates between elevated motivational states ${(y}_{3})$ and depressed states ${(y}_{2})$, with manic and depressive phase durations ${t}_{5}$ and ${t}_{6}$, yielding a cycle period $T={t}_{5}+{t}_{6}$ (approximately 300 days in our simulations) (Goodwin 2007). For melancholia, the clinical course was segmented into onset (${t}_{2}\approx 28$ days, sustained depressed episode (${t}_{3}\approx$2*24 days*), and recovery, with nadirs mapped to a qualitative HDRS-equivalent amplitude (${y}_{2}\approx$−38) (Parker 1996, Taylor and Fink 2006). These values guided qualitative matching of model trajectories. Representative values (including opponent time constants of approximately ${\tau }_{b}\approx 60-100$ days) and full equations are provided in the Supplementary Material.

### Opponent process dynamics in health

**Fig. 3:**
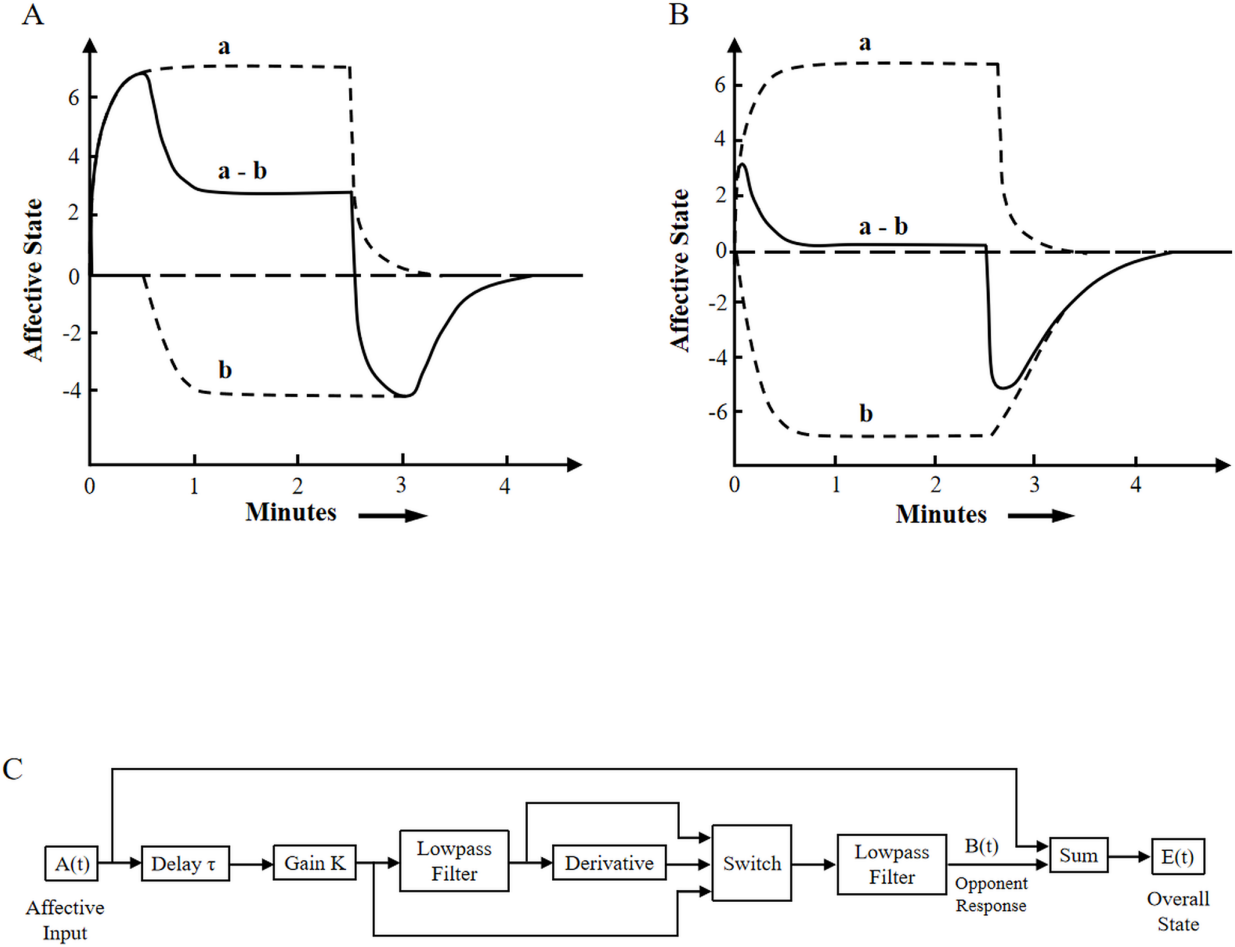
**A** Affective dynamics according to Solomon’s Opponent-Process Theory. Panel A illustrates affective responses to the initial presentation of a stimulus, with a prominent primary (**a**) process and a weaker opponent (**b**) process. Panel B shows the result of repeated stimulation: the a-process remains consistent, while the b-process becomes stronger, longer-lasting, and begins earlier. The resulting net affective response (**a** − **b**) exhibits reduced initial pleasure and a more pronounced negative after-reaction, reflecting adaptive emotional regulation over time. **B** Simulink implementation of the opponent-process model. The diagram presents a feed-forward control system simulating affective dynamics based on Solomon’s theory. The model includes an initial input (a-process), processed through delay, gain, low-pass filtering, and a derivative block. A switching mechanism activates the opponent b-process, which is further refined by additional filtering stages. This architecture enables simulation of both initial and adapted affective responses, capturing key features of emotional dynamics in health and across repeated exposures

Following Figs. 1 and 2, we implemented the opponent-process control model shown in Fig. 3. The model’s motivational trajectories in Fig. 3A, B closely reproduce Solomon and Corbit’s qualitative patterns in Fig. 1B–C. Consistent with their formulation, the model is feed-forward (Fig. 3C; cf. Figure 1D), with the net motivational state defined as the difference between the fast *a*-process and the delayed opponent *b*-process. A single stimulus produces a characteristic rise and post-stimulus opponent response (Fig. 3A). With repeated stimuli given after full recovery of the *b*-process, the *a*-process remains unchanged, whereas the *b*-process shifts earlier, increases in amplitude, and decays more slowly (Fig. 3B), reproducing the classical adaptive pattern. Model outputs reproduced Solomon and Corbit’s dynamics.

### Opponent process formulation

The net affective state was defined as the difference between the primary *a*-process and the opponent *b*-process:

$$e\left(t\right)=a\left(t\right)-b\left(t\right).$$

A finite stimulus of amplitude $A$, onset ${t}_{0}$, and duration $d$was modelled as (Fig. 2A):

$$a\left(t\right)={A}_{0} \left[u\left(t-{t}_{0}\right)-u\left(t-{t}_{0}-d\right)\right]$$

where $u(\cdot )$is the unit step function.

The opponent *b*-process was generated by a delayed, scaled, low-pass filtered transformation of $a\left(t\right)$:

$$b\left(t\right)=K\left[\right({h}_{1}*{h}_{2}*a\left)\right(t-{\tau }_{d}\left)\right]$$

or a Laplace-domain form:

$$B\left(s\right) = K{e}^{-{\tau }_{d}s} \cdot \frac{1}{(1+{\tau }_{1}s)(1+{\tau }_{2}s)}A\left(s\right)$$

with $K$ the opponent gain, ${\tau }_{d}$the onset delay, and ${\tau }_{1},{\tau }_{2}$the time constants of the adaptive stages, see Supplementary Material.

The stimulus was identical in Fig. 3A, B; adaptation was produced solely by varying the opponent parameters. In Panel A, a single exposure yields the classical Solomon trajectory. In Panel B, repeated exposures after full recovery of the *b*-process produce earlier onset, larger amplitude, and prolonged decay of $b\left(t\right)$, consistent with hedonic adaptation. The two-stage opponent pathway yields a smoother and longer post-stimulus opponent tail.

### Addiction as exogenous allostatic drift

Repeated exposure to a stimulus before recovery of the opponent *b*-process produces a progressive downward shift in affective state, a pattern Koob termed *allostasis* and extensively characterised in addiction physiology (Koob 2013). We used this phenomenon as a calibration step to verify that the model reproduces known allostatic dynamics before applying it to endogenous SEPI.

The net affective state was again defined as:

$$e\left(t\right)=a\left(t\right)-b\left(t\right)$$

A train of stimuli of amplitude $A$ and spacing ${\Delta }t$ was delivered before the *b*-process had returned to baseline. The opponent component was modelled using the same structure as in the healthy simulations, where ${\tau }_{s}$ represents the short-term withdrawal-like response and ${\tau }_{l}$ the slow allostatic accumulation, resulting in overlapping negative opponent responses and a progressive downward drift in affective state, see Supplementary Material:

$$b\left(t\right)={K}_{s}\cdot LP{F}_{{\tau }_{s}}\left[a(t-{\tau }_{d})\right]+{K}_{l}{\cdot LPF}_{{\tau }_{l}}\left[ a\left(t-{\tau }_{d}\right)\right]$$

or a Laplace-domain form:

$$B\left(s\right)=\left(\frac{{K}_{s}}{1+{\tau }_{s}s}+\frac{{K}_{l}}{1+{\tau }_{l}s}\right) {e}^{-{\tau }_{d}s}A\left(s\right)$$

**Fig. 4:**
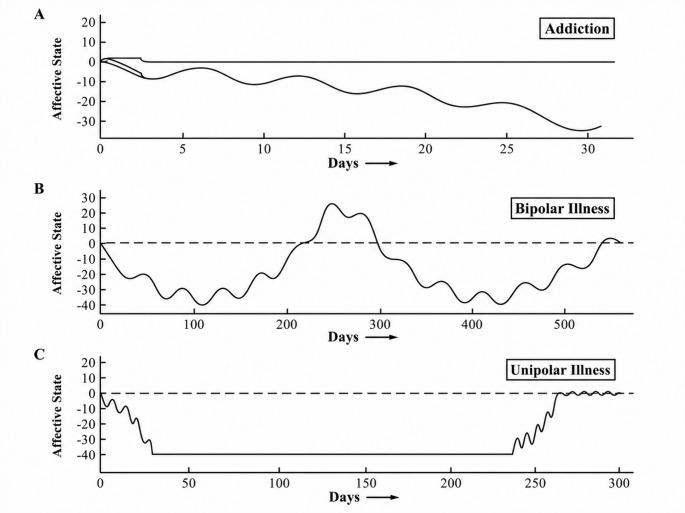
Simulated affective trajectories generated using a feed-forward control system implemented in Simulink, based on Solomon and Corbit’s Opponent-Process Theory. The model simulates the temporal interaction between the primary affective response (a-process) and the opponent response (b-process), using interpolated time-series inputs to drive system dynamics. A low-pass Butterworth filter and derivative-based control capture adaptation and homeostatic feedback. These plots illustrate how changes in parameter values (gain, delay, filter time constants) produce distinct clinical affective patterns: **A** Addiction: Repeated stimuli lead to increasing opponent activation and a progressive downward shift in baseline affective state (allostatic drift). **B** Bipolar disorder: Affective state oscillates over time, simulating alternating episodes of depression and mania, consistent with clinical mood cycling. **C** Unipolar depression: The model produces a sustained, pathological reduction in affective state, followed by a slow recovery, mimicking the time course of major depressive episodes. These simulated results reproduce key clinical patterns seen across affective disorders by modifying the temporal and amplitude characteristics of the opponent process. Toward baseline, reflecting typical clinical patterns observed during depressive episodes

Under these conditions the opponent response builds progressively sooner, reaches larger negative amplitudes, and decays more slowly after each exposure, producing a gradual downward drift of $e\left(t\right)$ over days (Fig. 4A). This reproduces Koob’s allostatic drift without changes to the model architecture and confirms that cumulative negative affect (which Koob terms hyperkatifeia) emerges naturally from repeated stimulation within the same homeostatic system.

### Melancholia as endogenous drift

Recurrent unipolar depression was modelled by assigning the opponent *b*-process a markedly increased gain and prolonged decay, while keeping the primary *a*-process identical to that used in the health simulations. Thus, normal cognitive–perceptual events produce a disproportionately large and slow opponent response. This generates an endogenous downward drift in the affective state, analogous in form to allostatic drift in addiction models but arising here from intrinsic abnormality of the opponent b-process instead of repeated exogenous drugs.

Clinical benchmarks were reproduced by tuning the opponent parameters so that the trajectory exhibited: an onset period of approximately 28 days, a sustained depressed phase of roughly 224 days, a nadir corresponding to HDRS-equivalent amplitude (≈ − 38), and a slow recovery toward baseline (Fig. 4C). These month-scale excursions follow directly from the prolonged time constant of the opponent system (${\tau }_{b}$ ≈ 60–100 days).

The net affective state was:

$$e\left(t\right)=a\left(t\right)-b\left(t\right)$$

This formulation follows Solomon’s definition and provides the basis for examining how changes in the opponent process alter the long-term trajectory of the affective state. The primary *a*-process was modelled identically across all conditions, including health, melancholia and bipolar illness. It was implemented as a finite-duration input of amplitude $A$, representing the immediate, stimulus-linked affective response:

$$a\left(t\right)=A\cdot u(t-t_{0})$$

In the unipolar simulations, no reduction of $A$ was introduced although it may be blunted and the abnormal trajectory arises from an increased opponent *b*-process (Suveges et al. 2025).

$${\tau }_{b}\frac{db\left(t\right)}{dt}+b\left(t\right)=K\cdot a(t-{\tau }_{d})$$

or a Laplace-domain form:

$$B\left(s\right)=K\cdot \frac{{e}^{-{\tau }_{d}s}}{1+{\tau }_{b}s}\cdot A\left(s\right)$$

With $K>1$and ${\tau }_{b}$in the 60–100-day range, the opponent response dominates the net trajectory, generating a prolonged downward deviation corresponding to the onset, maintenance, and gradual recovery phases of a depressive episode. Because ${\tau }_{b}$ is long, repeated everyday cognitive events occur before full recovery of $b\left(t\right)$, producing an endogenous drift analogous in form to allostasis but arising without repeated exogenous stimuli.

### Bipolar illness as endogenous oscillation

Bipolar illness was modelled as an intrinsic oscillatory instability within the same motivational homeostatic controller used for the healthy and unipolar simulations. The system exhibits spontaneous fluctuations between elevated and depressed motivational states. This corresponds to a classical underdamped second-order homeostatic failure mode, in which the regulatory mechanism overcorrects and reverses direction, producing alternating phases of mania/hypomania and depression. Consistent with clinical observations, the simulations were tuned to produce a cycle period of approximately 300 days, with shorter elevated phases and longer depressed phases, matching the typical asymmetry of bipolar trajectories (Fig. 4B).

The net affective state was:

$$e\left(t\right)=a\left(t\right)-b\left(t\right).$$

The *a*-process, representing the fast, stimulus-locked component of the affective response, was defined in the same way as in the healthy and unipolar simulations:

$$a\left(t\right)=A\cdot u(t-t_{0})$$

This produces endogenous alternation between elevated and depressed motivational states even in the absence of external perturbations.

$$\ddot{e}\left(t\right)+2\cdot \zeta \cdot \omega_{0}\cdot \ddot{e}\left(t\right)+{\omega_{0}^{2}}\cdot e\left(t\right)=0$$

The solution for the underdamped system has the standard oscillatory form with exponentially decaying envelope:

$$e\left(t\right)=E\cdot {e}^{(-\zeta \cdot \omega_{0}\cdot t)}\cdot cos({\omega }_{d}\cdot t+\varphi )$$

where:

$${\omega }_{d}=\omega_{0}\cdot \sqrt{1-\zeta^{2}}$$

The amplitude $E$ and phase $\varphi$ were chosen so that the peaks and troughs matched the typical clinical ranges of mania/hypomania and depression. The exponential term determines long-term stability; the cosine term generates alternating phases. The period of the bipolar cycle was determined by the damped angular frequency:

$$T=\frac{2\pi }{{\omega }_{d}}$$

Setting $T\approx 300$days reproduced the long-period oscillations characteristic of bipolar illness. The asymmetry between shorter elevated phases and longer depressed phases was achieved by selecting initial conditions and amplitude consistent with observed longitudinal data.

From a control-theoretic perspective, the revised model exhibits three qualitative regimes. When opponent gain is modest and recovery time constants are finite, the effective modes remain sufficiently damped and the output returns to baseline after stimulation, corresponding to stable homeostasis. Increasing opponent gain and prolonging recovery shifts the dominant mode toward a slow negative excursion, producing drift without oscillation. Reducing damping in the second-order representation yields complex conjugate modes with long period, producing endogenous underdamped oscillation. The difference between melancholia and bipolar illness therefore lies in the stability class of the same controller—slow drift versus oscillation—rather than in a change of architecture.

## Discussion

This proof-of-principle study tested whether Solomon’s opponent-process formulation can be expressed as a quantitative homeostatic controller of motivation, and whether affective illnesses can be interpreted as canonical failures within this system. The model reproduced normal a–b dynamics and, with simple parameter changes, generated two long-timescale clinical trajectories: a prolonged downward drift modelling melancholia and an underdamped oscillation modelling bipolar illness. These results should be interpreted as demonstrating dynamical plausibility within a minimal controller rather than as a uniquely fitted account of individual patients.

The drift–oscillation distinction offers a mechanistic interpretation of empirical findings rather than a one-to-one explanation of all clinical variance. In our recent study (Suveges et al. 2025). Unipolar melancholia and bipolar illness showed overlapping abnormalities in value learning and loss processing but diverged in reward-related signals. Melancholia displayed blunted striatal reward prediction errors and heightened hippocampal responses to aversive outcomes, whereas bipolar illness showed preserved striatal reward responses with greater lateral orbitofrontal sensitivity to loss. The present model is consistent with this pattern: an enlarged slowly recovering opponent process produces drift consistent with melancholia, whereas reduced damping preserves a-process reward-linked responses yet destabilises the controller sufficiently to generate an oscillatory course of bipolar illness. Thus both conditions reflect distinct dynamical regimes of a shared regulatory system.

The parsimony of the model is intentional and necessarily reductive. Melancholia and bipolar illness are heterogeneous syndromes shaped by developmental history, pharmacological exposure, circadian and endocrine modulation, and multi-scale neurobiology (Parker 1996, Taylor and Fink 2006, Goodwin 2007, Mizrachi et al. 2023, Hall and Hall 2021). Our claim is not that these factors are unimportant, but that diverse influences may converge on a smaller number of dynamical control parameters. In this sense, the model is best viewed as a parsimonious systems-level framework rather than a comprehensive disease ontology.

Interpreting affective illness as a failure of homeostatic control situates these conditions within classical physiology. In the Bernard–Cannon–Guyton framework (Hall and Hall 2021). Health reflects stable regulation of internal variables, while drift and oscillation represent characteristic model of regulatory failures. Applied to affective–motivational processes, the same controller can generate normal dynamics, drift and oscillatory instability without requiring a different controller class for each disorder. This provides a coherent way to think about overlap between melancholia and bipolar illness while remaining consistent with dimensional approaches such as RDoC, which emphasise shared positive- and negative-valence systems across diagnoses (Cuthbert and Insel 2013).

Contemporary computational neuroscience has clarified short-term abnormalities in learning and inference (Suveges et al. 2025), but most models do not identify the physiological mechanisms for month-scale trajectories. The opponent-process controller complements these approaches by embedding fast computations within a slower homeostatic structure. Changes in prediction errors or loss sensitivity can therefore be interpreted as altered contributions of a- and b-process rather than as wholly independent deficits, linking short-term neural computations to long-term clinical dynamics.

A further issue concerns feedback. Although physiological controllers typically include feedback, Solomon’s architecture is intrinsically feedforward, with the b-process driven by the a-process rather than by the net affective output. Introducing direct within-episode feedback in systems with long delays and slow recovery generically produces oscillatory instability, as predicted by classical control theory, which would confound the drift regime observed in melancholia. We therefore retain a feedforward formulation as an effective reduced description of the system. However, feedback can be reintroduced at a slower timescale: temporal-difference prediction-error signals arise at fast (trial-scale) timescales but act to update opponent-process parameters across episodes, such that their cumulative effect is slow adaptation of the b-process. This separation of timescales allows adaptive regulation without destabilising the within-episode dynamics.

In this formulation, prediction-error signals do not directly regulate affective state within an episode, but instead drive gradual adaptation of opponent-process parameters such as gain, recovery time, and damping. This provides a mechanistic link between experimentally observed value-related signals and long-timescale affective dynamics, while preserving the stability properties of the feedforward controller.

The current simulations are also illustrative rather than empirically estimated. Parameters were tuned to reproduce canonical durations, amplitudes, and asymmetries, but were not fitted to longitudinal individual patient data. A related limitation is identifiability: different combinations of gain, delay, decay, forcing, and damping may yield partially similar trajectories when only coarse symptom time series are observed. These therefore define an equivalence class within a low-dimensional parameter space rather than a uniquely identifiable inverse solution. Future work should therefore use constrained parameter estimation and recovery analyses, ideally combining longitudinal mood ratings with behavioural tasks and neuroimaging of valence circuitry, to determine which parameters are both recoverable and clinically discriminative.

The present model does not yet explicitly capture mixed states, rapid cycling, treatment effects, or state-dependent switching between episode types. These phenomena may correspond to intermediate or time-varying regions of parameter space, exogenous forcing, or treatment-induced changes in opponent gain and damping, but this remains speculative. Accordingly, the proposed biomarkers of opponent recovery time, delay, and damping should be regarded as candidate mechanistic markers that require empirical validation rather than clinically established measures.

Even with these limitations, the framework has practical significance. It provides a physiologically interpretable dynamical language for experimental design, hypothesis testing, and model-based stratification by linking shared affective circuitry to a small set of measurable quantities—opponent gain, recovery time, delay, and damping. Future work could integrate this controller with reinforcement learning, circadian-endocrine models, and direct parameter estimation from clinical, behavioural, and neuroimaging data.

More broadly, the model suggests that drift and oscillation may arise from different parameter settings within a shared motivational homeostat, offering a compact bridge between homeostatic physiology and computational psychiatry. This interpretation remains provisional, but it helps organize existing affective theories within a common dynamical framework and generates experimentally testable predictions for future work.

## Supplementary Information

Below is the link to the electronic supplementary material.

Supplementary Material 1.

Supplementary Material 2.

## Data Availability

No datasets were generated or analysed during the current study.
