# Supplementary material for: Cognitive neurodynamics of affective disorders
Source: Cogn Neurodyn. 2026 Jul 23;20(1):144. doi: 10.1007/s11571-026-10507-2 (PMC13396088; doi:10.1007/s11571-026-10507-2)
Supplement: Supplementary file 1 — Supplementary Material 1. [file 11571_2026_10507_MOESM1_ESM.pdf]

Supplementary Materials

Health

Computational Model of Affective Dynamics (a-b)

Mathematical Model

This section describes the affective dynamics modelled in the control block diagram shown in Fig. 1. The model is based on Solomon and Corbit's Opponent-Process Theory, where the observable affective state e(t) arises from the interaction between a primary process a(t) and an opponent process b(t).

We define the **affective state** over time as the interaction of two components:

- $a(t)$: primary emotional response (stimulus-driven, fast, fixed shape)
- $b(t)$: opponent response (delayed, slower, adaptive)
- $e(t)$: overall affective state = $a(t) - b(t)$

Primary Process (a-process)

This is the direct input from the stimulus, represented by the leftmost block, $A(t)$ $a(t)$

$$= A_0 u(t - t_0)$$

Where:

- A_0 is the amplitude of the stimulus
- $u(t - t_0)$ is the unit step function starting at = t_0

This signal passes unchanged to the summing block (final output), and also through the lower branch that generates the b-process.

Opponent Process (b-process)

This process is generated from $a(t)$ by a sequence of operations shown in the lower branch of the block diagram:

a. Delay:

$$a_d(t) = a(t - \tau_d)$$

- τ_d: physiological delay before b-process initiates

b. Gain Block:

$$a_g(t) = K a_d(t)$$

- K: scaling factor (typically positive to produce opponent effect)

c. Low-pass filtering (first stage):

$$b_1(t) = \frac{1}{\tau_1 s + 1} a_g(t)$$

τ_1: time constant of emotional adaptation (shorter = faster response)

d. Derivative Block

Captures sharp changes (optional, may be included for realism)

$$b_d(t) = \frac{d}{dt} b_1(t)$$

These models withdrawal effects or onset/offset dynamics.

e. Low-pass filtering (second stage):

The signal is then modulated based on context (repetition, duration, etc.) and passed to a second low-pass filter:

$$b(t) = \frac{1}{\tau_2 s + 1} b_d(t)$$

τ_2: longer adaptation memory (e.g., opponent process after many stimuli)

3. Overall Affective State

The final affective output is computed as:

$$e(t) = a(t) - b(t)$$

This equation corresponds to the $a - b$ curves shown in the top plots of Panels A and B, representing the net emotional state perceived by the individual.

Summary of Model Components

Block	Equation Component	Description
A(t)	$a(t) = A_0 u(t - t_0)$	Primary stimulus input
Delay (τ)	$a_d(t) = a(t - \tau_d)$	Physiological delay
Gain (K)	$a_g(t) = K a_d(t)$	Scaling of delayed input
Low-pass Filter 1	$B_1(s) = \frac{1}{\tau_1 s + 1} A_g(s)$	First-stage adaptation
Derivative	$b_d(t) = \frac{d}{dt} b_1(t)$	Captures withdrawal/transition
Low-pass Filter 2	$B_2(s) = \frac{1}{\tau_2 s + 1} B_d(s)$	Final opponent process
Summing Block	$e(t) = a(t) - b(t)$	Net affective state

Illness

Addiction

Addiction is characterized clinically by progressive changes in affective states associated with repeated exposure to addictive substances or behaviours. Initially, there are brief periods of heightened positive affect due to substance use, followed by increasingly prolonged and intense negative affective states during withdrawal. Over time, the baseline affective state progressively declines, reflecting the escalation of negative emotional states typical in chronic

addiction. This phenomenon represents allostasis, a process by which the body attempts to achieve stability through physiological or behavioural change in response to chronic stressors, ultimately altering the baseline set point of emotional regulation.

Computational Model

To simulate the progressive affective decline observed in addiction, we implemented a modular Simulink model that reflects the opponent-process theory adapted to an allostatic framework. The model captures both the acute hedonic effects of substance exposure and the delayed, accumulating negative affective states associated with withdrawal and chronic use.

The input signal is generated via a 1-D Akima interpolator, representing a smooth timedependent stimulus (e.g., drug administration). This input is processed through a discrete delay block (z^{-1}), followed by a gain block with a negative coefficient, which models an inhibitory or rebound-like effect. The signal then passes through a second-order low-pass filter to eliminate high-frequency noise, mimicking physiological constraints on emotional regulation. Subsequently, a derivative block captures rapid changes in affective state, and two b-process components model the delayed opponent response:

- The first b-process is a low-pass filter representing short-term withdrawal-like adaptation.
- The second b-process acts as a slow integrator, capturing cumulative negative affect and long-term downregulation of baseline mood.

These components are summed to yield the overall affective state (a + b), which is visualized through scope blocks. Over time, the system demonstrates increasing dominance of the negative b-process, consistent with the allostatic downward shift in emotional baseline observed in chronic addiction.

The study utilized MATLAB’s toolbox Simulink to construct dynamic control system models based on Solomon’s theory. Key variables (gain, delay, amplitude) were tuned to simulate normal emotional responses and pathological conditions. The simulations focused on two scenarios representing initial and repeated emotional stimuli, reflecting changes in emotional regulation and adaptation.

Addiction Modelling

Let:

- $a(t)$: primary process, fast, constant amplitude; • $b(t)$: opponent process, slow, adaptive, builds over time,
- $e(t) = a(t) - b(t)$: net affective state.

All models are built using:

- Delays: $a_d(t) = a(t-\tau)$
- $LPF_\tau[x(t)] = \frac{1}{1+\tau_s} x(t)$
- Nonlinear gain or switch blocks for state changes. Addiction model is described by the following equations:

$$a(t) = A_o u(t - t_0)$$

$$b(t) = LPF_{\tau 1}\{LPF_{\tau s}[K\, a(t - \tau_d)]\}$$

$$e(t) = a(t) - b(t)$$

- LPF_1: models acute withdrawal (short time constant)
- LPF_2: models long-term allostatic load (slow decay)

This produces a slow, progressive decline in $e(t)$, consistent with clinical allostasis.

Unipolar Modelling

With blunted a-processes small stochastic inputs are necessary to engage the slow opponent dynamics.

Assume an initial stimulus or disturbance:

$$a(t) = A_0\, u(t - t_0)$$

- A_0: amplitude ((can be small or even 0 to simulate blunted response)
- $u(t - t_0)$: step function at onset

The opponent process is defined by a strong gain and very slow filter:

$$B(s) = e^{-\tau_d s} \frac{K}{\tau_b s + 1} A(s)$$

or in time domain (first-order low-pass filter):

$$\frac{db(t)}{dt} = \frac{Ka(t - \tau_d) - b(t)}{\tau_b}$$

- $K \gg 1$:strong b-process amplification
- $\tau_b \gg 1$:long time constant (e.g.,60-90 days)
- τ_d:delay (typically 1-2 days)

$$e(t) = a(t) - b(t)$$

This results in:

- A steep drop in $e(t)$ during the onset phase (t_2)
- Sustained negative values during the depressive phase (t_3)
- Slow upward trend during recovery (t_4)

**Parameter**	**Description**	**Typical Value**
(y_2)	Depressive nadir/figure-axis scaling of $e(t)$; not a dynamic model parameter	−38 (Hamilton scale)
(t_2)	Onset and recovery durations	28 days each
(t_3)	Duration of depressive phase	224 days
(τ_b)	Opponent filter time constant	~60–100 days
(K)	Opponent gain	1.5–2.5×

Bipolar Modelling

No constant stimulus — instead, mood oscillates. A second-order underdamped system models spontaneous cyclic shift. The continuous-time formulation is a phenomenological approximation of slow episode-indexed adaptation dynamics in the limit of dense episodes, second-order oscillator:

$$\ddot{e}(t) + 2\zeta\omega\dot{e} + \omega^2 e(t) = 0$$

$\zeta < 1$: underdamped (oscillations)

$\omega = \frac{2\pi}{T}$, with $T \approx 300\ days$

Solution:

$$e(t) = A\, e^{-\zeta\omega t} \cos(\omega t + \phi)$$

- $a(t)$: can be zero or represent background noise
- $b(t)$: not explicitly separated, the dynamics are internal to $e(t)$

Panel C: Unipolar Depression

Initial stimulus or internal trigger causes a-process. b-process dominates and is very slow to decay.

Shows slow recovery to baseline after a prolonged negative state.

$a(t) = A_0\, u(t - t_0)$
$b(t) = LPF\ [K\ a(t - \tau)]$, with τlarge

$$e(t) = a(t) - b(t)$$

Here, $K \gg 1$, and $\tau \approx 60 - 100\ days$

This models long-lasting suppression of mood

Temporal Difference Learning

Note: the following is not part of the computational model presented in the manuscript. It is included to indicate how a physiologically-grounded learning signal could in principle formalise the between-episode changes in b-process parameters that underlie the clinical trajectories described above.

The model above captures within-episode affective dynamics under fixed opponent-process parameters. Solomon's theory has the b-process changing across repeated episodes: with experience it becomes greater in amplitude, faster to onset, and slower to resolve. We propose that this cross-episode adaptation can be formalised using temporal-difference (TD) learning;

the reinforcement-learning framework whose prediction-error signal is widely identified with phasic dopamine activity.

We propose that after each affective episode (n), the brain computes an affective return R_n (the time-integral of the within-episode affective state e(t) over the episode duration T_n: more positive values indicate net positive affect, more negative values indicate net negative affect), and a prediction error (*R.S. Sutton and A.G. Barto. Reinforcement Learning: An Introduction. 2nd ed. Cambridge, MA: MIT Press, 2018. ISBN 978-0262039246.*):

$$\delta_n = R_a + \gamma \cdot V_{(S_n+1)} - V_{(S_n)}$$

where S_n is the pre-episode affective/adaptive state and $S_n + 1$ is the post-episode state. $V_{(S_n)}$ is the system's prediction of affective outcome prior to episode n $\gamma \in (0, 1]$ is a discount factor weighting future episodes δ_n is the signed discrepancy between predicted and actual outcome; positive when outcomes exceed expectation, negative when worse. This prediction error updates the value estimate (*R.S. Sutton and A.G. Barto. Reinforcement Learning: An Introduction. 2nd ed. Cambridge, MA: MIT Press, 2018. ISBN 978-0262039246.):*

$$V_{(S_n)} \leftarrow V_{(S_n)} + \alpha_v \delta_n$$

Where $\alpha_v > 0$ is the value learning rate. This causes between-episode changes in the three bprocess parameters described above (opponent gain K, onset delay τ, and recovery time constant τ^b):

$K_{n+1} = K_n - \alpha^K \cdot \delta_n$ (opponent gain)

$\tau_{n+1} = \tau_n + \alpha\tau \cdot \delta_n$ (onset delay)

$\tau^{bn+1} = \tau^{bn} - {\alpha_t}^b \cdot \delta_n$ (recovery time constant)

The signs are chosen so that sustained negative prediction errors (outcomes chronically worse than expected) drive K upward, τ downward, and τ^b upward; producing an opponent process that is stronger, earlier in onset, and more prolonged. This is consistent with the progressive bprocess dominance observed in addiction and melancholia.

This framework accounts for both melancholia and bipolar illness trajectories described in the manuscript. In melancholia, prediction errors are chronically negative: K and τ^b accumulate across episodes while τ decreases, so the opponent term comes to dominate progressively and

affective baseline drifts downward. In bipolar illness, the same update rules produce oscillation when learning rates are large relative to the b-process time constants: opponent parameters overshoot before their full consequences occur, driving the system alternately into excessive opponent dominance (depression) and overshoot from euthymic baseline (mania). Both trajectories thus could in principle emerge from the same equations, differing only in learning rate magnitude, without requiring separate dynamical mechanisms. Model-based fMRI using temporal difference learning, such as we recently described (e.g. *Suveges et al 2025, Brain, doi:10.1093/brain/awaf280*) could be used to test this proposal.
