# Supplementary material for: Cognitive neurodynamics of affective disorders
Source: Cogn Neurodyn. 2026 Jul 23;20(1):144. doi: 10.1007/s11571-026-10507-2 (PMC13396088; doi:10.1007/s11571-026-10507-2)
Supplement: Supplementary file 2 — Supplementary Material 2. [file 11571_2026_10507_MOESM2_ESM.docx]

**Supplementary**

**1) Health**

**Computational Model of Affective Dynamics (a-b)**

# Mathematical Model

This section describes the affective dynamics modelled in the control block diagram shown in Fig. 1. The model is based on Solomon and Corbit’s Opponent-Process Theory, where the observable affective state e(t) arises from the interaction between a primary process a(t) and an opponent process b(t).

We define the **affective state** over time as the interaction of two components:

- $a\left( t \right)$: primary emotional response (stimulus-driven, fast, fixed shape)
- $b\left( t \right)$: opponent response (delayed, slower, adaptive)
- $e\left( t \right)$: overall affective state = $a\left( t \right)-b\left( t \right)$

## 1. Primary Process (a-process)

This is the direct input from the stimulus, represented by the leftmost block, $A\left( t \right)$

$$a\left( t \right)=A_{0}u(t-t_{0})$$

Where:

- $A_{0}$ is the amplitude of the stimulus
- $u(t-t_{0})$ is the unit step function starting at = $t_{0}$

This signal passes unchanged to the summing block (final output), and also through the lower branch that generates the b-process.

## 2. Opponent Process (b-process)

This process is generated from $a\left( t \right)$ by a sequence of operations shown in the lower branch of the block diagram:

a. Delay:

$$a_{d}\left( t \right)=a(t-\tau)$$

- $\tau$: physiological delay before b-process initiates

b. Gain Block:

$$a_{g}\left( t \right)=Ka_{d}(t)$$

- $K$: scaling factor (typically negative to produce opponent effect)

c. Low-pass filtering (first stage):

$$b_{1}\left( t \right)=\frac{1}{\tau_{1}s+1}a_{g}(t)$$

$\tau_{1}$: time constant of emotional adaptation (shorter = faster response)

d. Derivative Block

Captures sharp changes (optional, may be included for realism)

$$b_{d}\left( t \right)=\frac{d}{dt}b_{1}(t)$$

These models withdrawal effects or onset/offset dynamics.

e. Low-pass filtering (second stage):

The signal is then modulated based on context (repetition, duration, etc.) and passed to a second low-pass filter:

$$b\left( t \right)=\frac{1}{\tau_{2}s+1}b_{d}(t)$$

$\tau_{2}$: longer adaptation memory (e.g., opponent process after many stimuli)

## 3. Overall Affective State

The final affective output is computed as:

$$e\left( t \right)=a\left( t \right)-b(t)$$

This equation corresponds to the $a-b$ curves shown in the top plots of Panels A and B, representing the net emotional state perceived by the individual.

## Summary of Model Components

| Block | Equation Component | Description |
| --- | --- | --- |
| A(t) | $a\left( t \right)=A_{0}u(t-t_{0})$ | Primary stimulus input |
| Delay (τ) | $a_{d}\left( t \right)=a(t-\tau)$ | Physiological delay |
| Gain (K) | $a_{g}\left( t \right)=Ka_{d}(t)$ | Scaling of delayed input |
| Low-pass Filter 1 | $b_{d}\left( t \right)=\frac{d}{dt}b_{1}(t)$ | First-stage adaptation |
| Derivative | $b_{d}\left( t \right)=\frac{d}{dt}b_{1}(t)$ | Captures withdrawal/transition |
| Low-pass Filter 2 | $b\left( t \right)=\frac{1}{\tau_{2}s+1}b_{d}(t)$ | Final opponent process |
| Summing Block | $e\left( t \right)=e\left( t \right)-b(t)$ | Net affective state |

**Addiction Illness**

Addiction is characterized clinically by progressive changes in affective states associated with repeated exposure to addictive substances or behaviours. Initially, there are brief periods of heightened positive affect due to substance use, followed by increasingly prolonged and intense negative affective states during withdrawal. Over time, the baseline affective state progressively declines, reflecting the escalation of negative emotional states typical in chronic addiction. This phenomenon represents allostasis, a process by which the body attempts to achieve stability through physiological or behavioural change in response to chronic stressors, ultimately altering the baseline set point of emotional regulation.

**Computational Model**

To simulate the progressive affective decline observed in addiction, we implemented a modular Simulink model that reflects the opponent-process theory adapted to an allostatic framework. The model captures both the acute hedonic effects of substance exposure and the delayed, accumulating negative affective states associated with withdrawal and chronic use.

The input signal is generated via a 1-D Akima interpolator, representing a smooth time-dependent stimulus (e.g., drug administration). This input is processed through a discrete delay block (z⁻¹), followed by a gain block with a negative coefficient, which models an inhibitory or rebound-like effect. The signal then passes through a second-order low-pass filter to eliminate high-frequency noise, mimicking physiological constraints on emotional regulation.

Subsequently, a derivative block captures rapid changes in affective state, and two b-process components model the delayed opponent response:

- The first b-process is a low-pass filter representing short-term withdrawal-like adaptation.
- The second b-process acts as a slow integrator, capturing cumulative negative affect and long-term downregulation of baseline mood.

These components are summed to yield the overall affective state (a + b), which is visualized through scope blocks. Over time, the system demonstrates increasing dominance of the negative b-process, consistent with the allostatic downward shift in emotional baseline observed in chronic addiction.

The study utilized MATLAB’s toolbox Simulink to construct dynamic control system models based on Solomon’s theory. Key variables (gain, delay, amplitude) were tuned to simulate normal emotional responses and pathological conditions. The simulations focused on two scenarios representing initial and repeated emotional stimuli, reflecting changes in emotional regulation and adaptation.

**2) Addiction**

Let:

- $a\left( t \right)$: primary process, fast, constant amplitude;
- $b\left( t \right)$: opponent process, slow, adaptive, builds over time,
- $e\left( t \right)=a\left( t \right)-b\left( t \right)$: net affective state.

All models are built using:

- Delays: $a\left( t \right)=a(t-\tau)$
- Low-pass filters: $LPF[\frac{1}{\tau s+1}x(t)]$
- Integrators for cumulative effect:$\int x\left( t \right)dt$
- Nonlinear gain or switch blocks for state changes.

Addiction model is described by the following equations:

$$a\left( t \right)=A_{o}\sum u(t-t_{i})$$

$$b\left( t \right)=LPF_{2}\left[ \int LPF_{1}\left( K a\left( t-\tau\right) \right)dt \right]$$

$$e\left( t \right)=a\left( t \right)-b(t)$$

- $LPF_{1}$: models acute withdrawal (short time constant)
- $LPF_{2}$: models long-term allostatic load (slow decay)

This produces a slow, progressive decline in $e\left( t \right)$, consistent with clinical allostasis.

**3) Unipolar**

With blunted a-processes small stochastic inputs are necessary to engage the slow opponent dynamics.

**Equations:**

Assume an initial stimulus or disturbance:

$$a\left( t \right)=A_{0} u(t-t_{0})$$

- $A_{0}$: amplitude ((can be small or even 0 to simulate blunted response)
- $u(t-t_{0})$: step function at onset

The opponent process is defined by a strong gain and very slow filter:

$$b\left( t \right)=\frac{K}{\tau_{b}s+1}a(t-\tau)$$

or in time domain (first-order low-pass filter):

$$\frac{db(t)}{dt}=\frac{1}{\tau_{b}}(Ka\left( t-\tau\right)-b\left( t \right))$$

- $K\gg1$:strong b-process amplification
- $\tau_{b}\gg1$:long time constant (e.g.,60-90 days)
- $\tau$:delay (typically 1-2 days)

$$e\left( t \right)=a\left( t \right)-b(t)$$

This results in:

- A steep drop in $e\left( t \right)$ during the onset phase ($t_{2}$)
- Sustained negative values during the depressive phase ($t_{3}$)
- Slow upward trend during recovery ($t_{2}$ again)

| **Parameter** | **Description** | **Typical Value** |
| --- | --- | --- |
| ($y_{2}$) | Max negative deviation | −38 (Hamilton scale) |
| ($t_{2}$) | Onset and recovery durations | 28 days each |
| ($t_{3}$) | Duration of depressive phase | 224 days |
| ($\tau_{b}$) | Opponent filter time constant | ~60–100 days |
| ( K) | Opponent gain | 1.5–2.5× |

### **4) Bipolar**

### **Panel B: Bipolar Illness**

### No constant stimulus — instead, mood oscillates.

A second-order underdamped system models spontaneous cyclic shift.

The continuous-time formulation is a phenomenological approximation of slow episode-indexed adaptation dynamics in the limit of dense episodes.

**Equation (second-order oscillator):**

$$\ddot{e}\left( t \right)+2\vartheta\omega\dot{e}+\omega^{2} e\left( t \right)=0$$

$\vartheta<1$: underdamped (oscillations)

$\omega=\frac{2\pi}{T}$, with $T\approx300 days$

Solution:

$$e\left( t \right)=A e^{-\vartheta\omega t}\cos\left( \omega t+\phi\right)$$

- $a(t)$: can be zero or represent background noise
- $b(t)$: not explicitly separated, the dynamics are internal to $e\left( t \right)$

### **Panel C: Unipolar Depression**

Initial stimulus or internal trigger causes a-process.

b-process dominates and is very slow to decay.

Shows slow recovery to baseline after a prolonged negative state.

**Equations:**

$$a\left( t \right)=A_{0} u(t-t_{0})$$

$\left( t \right)=LPF (K a(t-\tau$)), with $\tau$large

$$e\left( t \right)=a(t)-b(t)$$

Here, $K\gg1$, and $\tau\approx60-100 days$

This models long-lasting suppression of mood

**Computational Model of Affective Dynamics**

To quantitatively explore the dynamics of emotional regulation in health and psychiatric illness, we developed a control-theoretic model inspired by Solomon and Corbit’s Opponent-Process Theory. In this framework, emotional states emerge from the interplay between a fast-acting primary process $a\left( t \right)$), which captures the immediate hedonic response to a stimulus, and a slower opponent process $b\left( t \right)$, which evolves over time to counteract and normalize the affective state.

The overall observable affective state is defined as:

$$e\left( t \right)=a\left( t \right)-b\left( t \right)$$

where $a\left( t \right)$ is typically modelled as a step or pulse input representing an emotionally salient event, and $b\left( t \right)$is computed as a delayed, filtered, and scaled transformation of $a\left( t \right)$, incorporating both short-term adaptation and longer-term memory effects.

This opponent-process interaction was implemented in MATLAB Simulink as a modular feed-forward system, including delay elements, gain scaling, low-pass filters, and integrators to reflect neurophysiological constraints. The model parameters (e.g., gain, time constants) were adjusted to simulate distinct clinical conditions—addiction, bipolar disorder, and unipolar depression—by altering the temporal and amplitude characteristics of $b\left( t \right)$. Online with respect to experience, but not continuous-time feedback on affective state. These simulations reproduce key affective trajectories observed in clinical practice, including hedonic adaptation, cyclical mood oscillations, and sustained affective suppression.

**Unipolar Depressive Illness**

Severe unipolar depressive illness is characterized clinically by prolonged episodes of significantly reduced mood, motivation, and overall affective state. Patients experience a sustained negative deviation from their normal euthymic baseline lasting from weeks to months, followed by a gradual recovery phase back toward baseline. The pattern shown aligns with clinical observations of depressive episodes, highlighting both the depth and duration of affective dysregulation typically observed in severe cases.

**Computational Model:**

The unipolar depression model captures a three-phase affective trajectory commonly observed in major depressive episodes:

Stage 1 – Developing Illness: A decline in affect begins after an initial trigger.

Stage 2 – Depression: A prolonged period of low affective state, with limited recovery or fluctuation.

Stage 3 – Recovery: Gradual return to euthymic baseline as the opponent system is reduced.

This model emphasizes the persistence and depth of the negative emotional state, shaped primarily by a dominant and slow-decaying opponent b-process, which outweighs a weak or absent a-process. It can also simulate cases where the a-process is blunted or does not respond normally to positive stimuli.
